# BQ323636.1 Employs the AR-CCRK Axis to Modulate the Expression of KU70 to Interfere with Non-Homologous End Joining Mediated DNA Repair Mechanism †

**DOI:** 10.3390/cells14171341

**Published:** 2025-08-29

**Authors:** Ho Tsoi, Zi-Qing So, Ellen P. S. Man, Chan-Ping You, Koei Ho-Lam Cheung, Yin-Suen Tse, Wing-Lok Chan, Ui-Soon Khoo

**Affiliations:** 1 Department of Pathology, School of Clinical Medicine, Li Ka Shing Faculty of Medicine, The University of Hong Kong, Hong Kong, China; tsoiho@hku.hk (H.T.); u3569644@connect.hku.hk (Z.-Q.S.); u3006037@connect.hku.hk (C.-P.Y.); cheunghlk@connect.hku.hk (K.H.-L.C.); tyssuen@hku.hk (Y.-S.T.); 2 Department of Clinical Oncology, Li Ka Shing Faculty of Medicine, The University of Hong Kong, Hong Kong, China; winglok@hku.hk

**Keywords:** breast cancer, doxorubicin resistance, BQ323636.1, androgen receptor, CCRK, KU70, NHEJ

## Abstract

BQ323636.1 (BQ) is a splice variant of NCOR2. Its overexpression is associated with endocrine therapy and chemoresistance in estrogen receptor-positive (ER+ve) breast cancer. This study investigates how BQ overexpression drives doxorubicin (DOX) resistance by enhancing androgen receptor (AR) signaling and non-homologous end joining (NHEJ). BQ overexpressed breast cancer cell lines (MCF-7, T-47D, BT-549, MDA-MB-453), showed increased AR activity (ARE-luciferase assay) and demonstrated DOX resistance (EC50 > 10-fold with DHT, *p* < 0.05), as assessed via cell viability, TUNEL, and comet assays. RNA-sequencing (GSE295979, GSE2048) revealed the involvement of AR signaling. BQ upregulated cell cycle-related kinase (CCRK), stabilizing KU70, a key NHEJ protein, resulting in enhanced NHEJ activity (EJ5-GFP assay, *p* < 0.01). Co-immunoprecipitation confirmed the interaction between CCRK and KU70, and CCRK was found to modulate the protein stability of KU70. AR inhibition with bicalutamide in BQ overexpressing cells reversed DOX resistance. Xenograft models validated AR-dependent DOX resistance. In ER+ve breast cancer patient samples, high CCRK expression correlated with DOX resistance (*p* = 0.002) and metastasis (*p* = 0.001). Kaplan–Meier analysis showed poorer overall survival (*p* < 0.001) and disease-specific survival (*p* < 0.001) in cancers with high CCRK. Cox-regression analysis showed that high CCRK was a poorer prognostic factor of overall survival (*p* < 0.001; RR 3.056, 95% CI 1.661, 5.621, AR (*p* < 0.001; RR 3.420, 95% CI 1.783, 6.562), and disease-specific survival (*p* < 0.001; RR 2.731, 95% CI 1.472, 5.067). The BQ-AR-CCRK-KU70 axis represents a novel mechanism of DOX resistance in ER+ve breast cancer, suggesting AR or CCRK inhibition as a potential therapeutic strategy.

## 1. Introduction

Breast cancer is a heterogeneous disease with distinctive molecular subtypes: luminal A, luminal B, HER2-enriched, basal, normal-like, and claudin-low [1,2]. These subtypes guide prognosis and treatment decisions, with molecular tests such as Oncotype DX, PAM50, EndoPredict, and Breast Cancer Index used to assess recurrence risk and therapeutic strategies [3]. Treatment decisions primarily depend on the expression of receptor proteins: estrogen receptor-α (ER+ve), progesterone receptor (PR+ve), and human epidermal growth factor receptor 2 (HER2). ER+ve breast cancers are typically treated with endocrine therapies, such as tamoxifen, a selective estrogen receptor modulator (SERM), while HER2+ cancers receive anti-HER2 therapies [4]. Triple-negative breast cancers (TNBC), lacking these receptors, rely heavily on chemotherapy, often incorporating drugs like doxorubicin (DOX) to enhance treatment efficacy [5].

Drug resistance remains a significant challenge in breast cancer management. Complex molecular pathways, rapid cell proliferation, compounded by tumor heterogeneity, are among the factors that contribute to resistance [6]. Mutations and global alterations of gene expression profiles occur with cancer progression [7,8]. Some mutations, such as p53 [9], disrupt the function of tumor suppressors, whilst other mutations will make proteins involved in cell proliferation and constitutively activate survival pathways, such as PI3KCA [10] and AKT [11]. Unregulated PI3KCA and AKT have been shown to confer drug resistance, including chemotherapy, in breast cancer [12,13]. In prostate cancer, hyperactivation of androgen receptor (AR) signaling promotes tumorigenesis and castration-resistant disease, partly by enhancing DNA repair capacity [14,15,16,17]. Similarly, in breast cancer, AR signaling may modulate DNA repair, particularly in response to DNA-damaging agents like DOX, which induces lethal double-strand breaks (DSBs) if unrepaired [18,19]. DNA repair mechanisms, including non-homologous end joining (NHEJ) and homologous recombination (HR), mediated by proteins such as DNA-dependent protein kinase (DNA-PK) and RAD51, can reduce the efficacy of DOX [20]. It has been reported that AR can modulate the activity of DNA-PK and enhance the DNA repair capacity, making cancer cells tolerant to chemotherapy [17,21]. Notably, AR activation has been implicated in enhancing NHEJ and HR, potentially conferring resistance to chemotherapy [22,23,24]. Therefore, targeting AR should compromise DNA repair ability.

Given the high prevalence of AR (70–95%) in ER+ breast cancers and its context-dependent prognostic role, understanding the contribution of AR to DOX resistance is important [25,26,27]. The prognostic value of AR appears to be dependent upon ER status. It has been demonstrated that AR activation can suppress ER+ve breast cancer [28]. However, in ER-ve/HER2+ve breast cancer, AR could be oncogenic by regulating the levels of WNT7B and activating β-catenin, resulting in cell proliferation and tumor growth [29]. The effect of AR on DNA repair in ER+ve breast cancer, however, remains to be addressed, and it is unclear whether AR inhibition can compromise doxorubicin resistance in ER+ve breast cancer.

Our group identified a novel splice variant of NCOR2, BQ323636.1 (BQ) [30], and we confirmed its clinical significance in breast cancer [31], particularly that of endocrine resistance. Mechanistically, we showed BQ competes with NCOR2 to compromise the suppressive role of NCOR2 in regulating transcription of ER target genes [31], AR-responsive genes [32], and NRF2 transcriptional activity [33]. BQ overexpression can enhance ER signaling [31] and AR signaling [32], compromising the efficacy of tamoxifen and aromatase inhibitors, respectively. BQ overexpression through enhanced NRF2 transcriptional activity [33] was also associated with epirubicin resistance [33]. In this study, we hypothesized that BQ may employ AR to modulate DOX resistance. We found that AR mediated the expression of cell cycle-related kinase (CCRK; also known as CDK20). CCRK could modulate the protein stability of KU70, which is an essential protein for NHEJ. Knockdown of CCRK could reduce KU70 expression and, thus, the activity of NHEJ. On the other hand, BQ overexpression could enhance the KU70 protein level as well as NHEJ activity. In vivo studies confirmed that BQ was correlated with AR and CCRK expressions. High expression of CCRK was a poor prognostic factor and was associated with DOX resistance in ER+ve breast cancer.

## 2. Materials and Methods

### 2.1. Cell Culture, Transfection, siRNA, and Stable Cell Line Establishment

MCF-7 (HTB-22), T-47D (HTB-133), ZR-75-1 (CRL-1500), MDA-MB-231 (CRM-HTB-26), MDA-MB-436 (HTB-130), and MDA-MB-453 (HTB-131) were obtained from the American Type Culture Collection (Manassas, VA, USA). BT-20 (iCell-h441) and BT-549 (iCell-h029) were obtained from Cellverse Co., Ltd. (Shanghai, China). BQ overexpression pcDNA3.1-His-BQ (BQ OE) and the control pcDNA3.1-His (Ctrl OE) plasmids were employed [31]. Transfection was performed using Lipofectamine™ 2000 Transfection Reagent (11668027, Thermo Fisher Scientific, Waltham, MA, USA). Stable BQ-expressing cell lines were established by Geneticin™ Selective Antibiotic selection (200–500 µg/mL; 10131035, Thermo Fisher Scientific, Waltham, MA, USA). The cells were cultured in DMEM (12100046, Thermo Fisher Scientific, Waltham, MA, USA) supplemented with 10% FBS (26140079, Thermo Fisher Scientific, Waltham, MA, USA) and 1% P/S (10378016, Thermo Fisher Scientific, Waltham, MA, USA). The cell lines were kept in the incubator at 37 °C, and the incubator was supplied with 5% CO_2_. For the stable cell lines, 200 µg/mL of Geneticin™ Selective Antibiotic (10131035, Thermo Fisher Scientific, Waltham, MA, USA) was added to the culture medium. Small interference RNAs (siRNA) that target CCRK (sc-92544) and the non-targeting siRNA (siCtrl; sc-37007) were purchased from Santa Cruz Biotechnology (Dallas, TX, USA). Small-interference RNA was transfected using Oligofectamine™ transfection reagent (12252011, Thermo Fisher Scientific, Waltham, MA, USA). The optimal concentration, 50 nM, of the siRNA and siCtrl was used, and was performed according to instructions from the manufacturer.

### 2.2. Molecular Cloning

CCRK was amplified using the primers (5′ → 3′): BamH1_HA_CCRK_F, CGG GAT CCA TGT ACC CAT ACG ATG TTC CAG ATT ACG CTA TGG ACC AGT ACT GCA TCC TGG G and EcoR1_stop_CCRK_R, CGG AAT TCC TAG ACG CTG AGG AAT CGG CAG. High Fidelity Taq DNA Polymerase (11304-011, Thermo Fisher Scientific, Waltham, MA, USA) was employed. The PCR product was purified using Wizard^®^ SV Gel and PCR Clean-Up System (A9281, Promega Corporation, Madison, WI, USA). The purified PCR product and pcDNA3.1 were digested with BamH1-HF (R3136S, New England Biolabs, Ipswich, MA, USA) and EcoR1-HF (R3101S, New England Biolabs, Ipswich, MA, USA). The digested DNA was purified using Wizard^®^ SV Gel and PCR Clean-Up System (A9281, Promega Corporation, Madison, WI, USA). T4 DNA Ligase (M0202S, New England Biolabs, Ipswich, MA, USA) was employed to generate pcDNA3.1-HA-CCRK mammalian expression plasmid.

### 2.3. Chemicals

Doxorubicin (DOX; S1208, Selleck Chemicals LLC, Houston, TX, USA), Bicalutamide (BIC; S1109, Selleck Chemicals LLC, Houston, TX, USA), Dihydrotestosterone (DHT; S4757, Selleck Chemicals LLC, Houston, TX, USA), and MG132 (S2619, Selleck Chemicals LLC, Houston, TX, USA) were obtained. They were dissolved in a solvent: DMSO.

### 2.4. ARE-Luciferase Reporter Assay

Luciferase reporter assay with androgen-responsive element (CCS-1019L, Qiagen, Hilden, Germany) was employed to monitor the activity of AR. Dihydrotestosterone (DHT; Purity > 98% as confirmed by HPLC; S4757, Selleck Chemicals LLC, Houston, TX, USA) was dissolved in DMSO. Transfection was performed according to instructions from the manufacturer. The signal was captured by an Infinite F200 microplate reader (Tecan, Männedorf, Switzerland).

### 2.5. Cell Viability Assay

5000 to 10,000 cells were seeded in triplicate in a 96-well plate, and the cells were treated for 96 h. Doxorubicin (Dox; Purity > 99%; E2516, Selleck Chemicals LLC, Houston, TX, USA) and bicalutamide (Bic; Purity > 99%; S1190, Selleck Chemicals LLC, Houston, TX, USA) were dissolved in DMSO. Relative cell viability was determined by comparing to the untreated control. Cell viability was determined using CCK8 reagent (C0038, Beyotime Biotechnology, Haimen, China). The absorbance was recorded by an Infinite F200 microplate reader (Tecan, Seestrasse, Switzerland). EC50 value was calculated using GraphPad PRISM 10.1.2 through a non-linear regression model with [DOX] vs. cell viability (three parameters).

### 2.6. TUNEL Assay and Comet Assay

TUNEL assay (C1088, Beyotime Biotechnology, Haimen, China) was performed. 50,000 cells were seeded into 96 black well plates (165305, Thermo Fisher Scientific, Waltham, MA, USA). The signal was developed 48 h post-treatment. The fluorescence intensity was monitored and recorded using SpectraMax^®^ i3x Multi-Mode Microplate Reader (Molecular Devices, San Jose, CA, USA). Comet Assay Kit (C2041M, Beyotime Biotechnology, Haimen, China) was used according to the instruction manual. Nuclei were stained using 20 µg/mL of Hoechst 33342 (H1399, Thermo Fisher Scientific, Waltham, MA, USA). Electrophoresis (25 V for 20 min) was performed under alkaline conditions. The electrophoresis buffer (1 mM EDTA, 200 mM NaOH) was used. 100 nuclei were analyzed, and % of DNA in the tail was determined using OpenComet v1.3.1 [34].

### 2.7. RT-qPCR

Total RNA was isolated using Trizol reagent (15596026, Thermo Fisher Scientific, Waltham, MA, USA). 500 ng of the RNA was used for generating cDNA by reverse transcription (4368814, Thermo Fisher Scientific, Waltham, MA, USA). qPCR was performed, and the signal was generated by SYBR green in ROX-containing solution (A25741, Thermo Fisher Scientific, Waltham, MA, USA). The following primers (5′ → 3′) were used: KU70-F, TGC ACC TGA AGA AAC CTG GG; KU70-R, CCT TCC GCA ACA GGT CTT CT; KU80-F, CTG TGT ATG GAC GTG GGC TT; KU80-R, AGC AAA CAC CTG TCG CTG TA; PRKDC-F; CGT TTG GAT CCG CTA CAC AG PRKDC-R; GGC CCG TTT CTT TCA TTG GT; LIG4-F, GCA GAG ATC GTA CCC AGT GA; LIG4-R, GAT GCG AGC TTA CCA GAT GC; XLF-F, ATT CTA CGG GTG CGA AGT GA; XLF-R; CTC CCT CAC TTG GCA CTG TA; DCLRE1C-F, TGG GCT CTG TAC TTC ACC TG; DCLRE1C-R, ACT GTG GAG GAA GGG AAG T; LMTK3-F, CCT TCG TGG TTC AAG TGA GC; LMTK3-R, ACT TTC TCT CTG TTC CCG GG; GSK3β-F, TCC TCC TCA TGC TCG GAT TC; GSK3β-R, AGG TGG AGT TGG AAG CTG AT; CCRK-F, TTG TGC TGG CCT TTG AGT TC; CCRK-R, TGT TGT TGG CAT GGC AGA AG; MAPK4-F, ATG CTT ACG GGG AGA ATG CT; MAPK4-R, AGC TCG TCC TTG TCT TCC TC; MAP3K10-F, AGA AGG AAG AAC TGG TCG GG; MAP3K10-R, CAC TGT TTG CTT CCT TCC CC; ACTB-F, ACT CTT CCA GCC TTC CTT CC; ACTB-R, CGT ACA GGT CTT TGC GGA TG.

### 2.8. Western Blot

Cells were lysed using the lysis buffer (20 mM Tris-Cl, pH 7.4, 150 mM NaCl, 10% Glycerol, 0.1% SDS, 0.5% NP-40). Protein assay (5000112; Bio-Rad, Hercules, CA, USA) was performed, and 20 µg of protein was used in the Western blot. The proteins were separated using SDS-PAGE and transferred to the PVDF membrane. The following primary antibodies were used: anti-HIS tag (1:10,000; MA1-135, Thermo Fisher Scientific, Waltham, MA, USA); anti-HSP90 (1:5000; #4874, Cell Signaling Technology, Danvers, MA, USA), anti-HA (1:2000; #11846, Cell Signaling Technology, Danvers, MA, USA), anti-ATR (1:1000; #2790, Cell Signaling Technology, Danvers, MA, USA), anti-DNA-PKcs (1:4000; #4602, Cell Signaling Technology, Danvers, MA, USA), KU70 (1:1000; #4588, Cell Signaling Technology, Danvers, MA, USA), anti-CCRK (1:1000; ab252986, Abcam Limited, Cambridge Biomedical Campus, Cambridge, UK), anti-GAPDH (1:20,000; sc-166574, Santa Cruz Biotechnology, Dallas, TX, USA), anti-ubiquitin (1:5000; sc-166553, Santa Cruz Biotechnology, Dallas, TX, USA). The following secondary antibodies were used: anti-mouse HRP (1:5000; P0447, Agilent Dako, Santa Clara, CA, USA) and anti-rabbit HRP (1:5000; P0260, Agilent Dako, Santa Clara, CA, USA). The chemiluminescent signal was generated using BeyoECL Star (P0018AS, Beyotime Biotechnology, Haimen, China). Amersham Imager 680 (GE HealthCare, Chicago, IL, USA) was used to capture and record the signal. ImageJ from NIH was employed to measure the band intensity. Band intensity relative to GAPDH was determined.

### 2.9. Co-Immunoprecipitation

Cell lysates were prepared in 200 µL of IP buffer (20 mM Tris-Cl, pH 7.6, 100 mM NaCl, 5 mM MgCl_2_, 10% Glycerol, 0.5% NP-40, 0.5% sodium deoxycholate) supplemented with proteinase inhibitor cocktail (78429, Thermo Fisher Scientific, Waltham, MA, USA). The cell lysates were precleared with 50 μL of Protein A/G Magnetic Beads (PB101-01, Vazyme, Nanjing, China) on ice for 60 min. Protein concentration was determined using a protein assay (5000112, Bio-Rad, Hercules, CA, USA). 1 mg of proteins with volume adjusted to 500 μL with IP buffer was for the immunoprecipitation with anti-HA (1:100; #11846, Cell Signaling Technology, Danvers, MA, USA), anti-KU70 (1:100; #4588, Cell Signaling Technology, Danvers, MA, USA) or the control anti-rabbit IgG (1:100; 30000-0-AP, Proteintech Group Inc, Rosemont, IL, USA) at 4 °C overnight. The immunoprecipitation products were incubated with 50 μL of Protein A/G Magnetic Beads (PB101-01, Vazyme, Nanjing, China) for 2 h at 4 °C. The magnetic beads were washed with 1 mL of IP buffer for 10 min, and the step was repeated three times. The beads were then collected, and the proteins were eluted by incubating with 50 μL of 2× SDS sample buffer at 99 °C for 10 min. The samples were subjected to Western blot. Band intensity was determined using ImageJ (Version 1.54k). The ratio of elute to input in anti-IgG and anti-HA fractions was determined. For the poly-Ub assay on KU70, the ratio of KU70 elute to input was determined to obtain a normalized KU70. Then, the total poly-Ub signal in the elute was acquired by selecting the region with the smear and bands. Next, the ratio of total poly-Ub signal to normalized KU70 was determined, reflecting the relative amount of poly-Ub on KU70.

### 2.10. HR and NHEJ Assays

pDRGFP (#26475) [35], pimEJ5GFP (#44026) [36], and pCBASceI (#26477) [37] were obtained from Addgene (Watertown, MA, USA). 1 × 10^6^ cells were seeded into a 6-well plate, and the cells were transfected with 1 µg of pDRGFP or pimEJ5GFP together with 1 µg of pCBASceI. 48 h post-transfection, the cells were detached. 20,000 cells were seeded into a 96-well plate. Samples were triplicate. To ensure consistent transfection efficiency, identical amounts of plasmids were used across all control and experimental groups. This standardized approach was employed to compensate for the lack of direct normalization for transfected cell selection, as the plasmids used did not contain internal selection markers. The cells were treated with 1 nM of DHT or 5 µM of Bic for 48 h. GFP signal was recorded using a microplate reader Infinite F200 (Tecan, Seestrasse, Switzerland).

### 2.11. Gene Expression and Pathway Enrichment Analyses

Gene expression profiles from GSE2048 [38] and GSE295979 [39] were employed. Results were analyzed, and a heatmap was generated using iDEP 0.96 [40]. Pathway enrichment analysis was performed using the KEGG dataset [41]. Gene set enrichment analysis was employed as an independent method to determine the enrichment of the AR response [42]. The potential kinase was identified using the built-in function of iDEP 0.96.

### 2.12. Xenograft

The work was approved by the HKU Committee on the Use of Live Animals in Teaching and Research (CULATR 23-571) on 15 Jan 2024. Six-week-old to eight-week-old female BALB/cAnN-nu nude mice were used. 1 × 10^7^ cells of MCF-7 and MDA-MB-453 in 75 μL of PBS mixed with 75 μL of Matrigel from Corning (356234; BD Bioscience, Franklin Lakes, NJ, USA). The cell mixture was implanted into the mammary fat pad. Doxorubicin (DOX; S1208, Selleck Chemicals LLC, Houston, TX, USA). DOX was dissolved in DMSO to prepare a stock solution. Then, a solvent was prepared by adding 50 μL of a 100 mg/mL DOX stock solution to 400 μL of PEG 300 (S6704, Selleck Chemicals LLC, Houston, TX, USA), and the mixture was mixed evenly to clarify it. Then, 50 μL of Tween80 (S6702, Selleck Chemicals LLC, Houston, TX, USA) was added. Next, 500 μL of ddH2O was added to adjust the volume to 1 mL. The mixed solution should be used immediately for optimal results. 5.0 mg/mL of DOX solution was prepared. Dihydrotestosterone (DHT; S4757, Selleck Chemicals LLC, Houston, TX, USA) was employed and dissolved in DMSO. 100 μL of 50 mg/mL DHT stock solution was added to 400 μL of PEG300 (S6704, Selleck Chemicals LLC, Houston, TX, USA). Then, 50 μL of Tween80 (S6702, Selleck Chemicals LLC, Houston, TX, USA) was added. Subsequently, 450 μL ddH2O was added to adjust the volume to 1 mL. The mixed solution should be used immediately. The mice were treated with 15 mg/Kg of DOX and 12.3 mg/Kg of DHT via subcutaneous injection. The treatment was performed twice per week. The largest and smallest diameters of the tumor were measured using an electronic caliper. The volume of the subcutaneously xenografted tumors was determined [39].

### 2.13. Immunohistochemistry and TMA

Clinical investigation was approved (HKU/HA HKW IRB No. UW 08-147) by the Institutional Review Board of The University of Hong Kong and Hospital Authority Hong Kong West Cluster on 16 February 2024. A total of 170 breast cancer cases (Table 1) were included in the current study. Patients diagnosed with primary ER+ve breast cancer, who had received doxorubicin therapy, either in the neoadjuvant or adjuvant setting, with at least 5 years of clinical follow-up information, were retrieved from the files of the Department of Pathology, Queen Mary Hospital, Hong Kong SAR, China. Histological sections were analyzed and confirmed by the pathologist. Donor blocks were chosen from the representative paraffin tumor blocks of each case, and selected regions were marked for the construction of Tissue MicroArray (TMA). DOX resistance was defined as patients treated with DOX who initially responded but subsequently developed disease recurrence or distant metastases. Only cases with a clear history of treatment response were used for the analysis of DOX resistance. TMA sections were deparaffinized using xylene, and the sections were rehydrated with ethanol in a series of decreasing concentrations from 100% to 70%. Antigen retrieval process was performed using 1× SSC buffer (pH 6.0). The slides were then incubated in 3% H_2_O_2_ solution to quench endogenous peroxidase. The slides were rinsed with 1× TBST three times, followed by incubation with primary monoclonal anti-CCRK antibody (1:50; ab252986, Abcam Limited, Cambridge Biomedical Campus, Cambridge, UK) at 4 °C overnight. BQ [31] and AR [32] were stained using the previously established protocols. The slides were washed three times with 1× TBST and incubated with secondary anti-rabbit (K4003; Agilent Dako, Santa Clara, CA, USA) or anti-mouse (K4001; Agilent Dako, Santa Clara, CA, USA) for 30 min at room temperature. The slides were then washed three times with 1× TBST, followed by incubation with chromogen DAB/substrate reagent (K3467, Agilent Dako, Santa Clara, CA, USA) for 5 min. After dehydration with ethanol and xylene, the slides were mounted. Images were captured using the Nanozoomer system S210 microscopic slide scanning system (Hamamatsu Photonics K.K., Hamamatsu City, Shizuoka, Japan). Two individuals performed the scoring. H-score was calculated: 1 × % of cells stained at low intensity + 2 × % of cells stained at moderate intensity + 3 × % of cells stained at high intensity. The median of the H-score was used as the cut-off, which was 150 for nuclear BQ, 105 for nuclear AR, and 120 for nuclear CCRK. High expression was regarded as having an H-score greater than the median.

### 2.14. Statistical Analysis and Graphic Illustration

All data were recorded, processed, and stored in Excel (Office365, Microsoft, Redmond, WA, USA), Prism10 (GraphPad Software, Boston, MA, USA), or SPSS25 (IBM, Armonk, NY, USA). Mean ± SD was used to show the results. Student’s *t*-tests were performed to determine the significance between the 2 groups. One-way and Two-way ANOVA with Bonferroni’s post-test was employed for multiple group comparisons. Chi-square (χ^2^) test was employed to reject the null hypothesis. The Pearson coefficient was employed to determine the correlation. The survival analyses were performed using Kaplan–Meier estimates with the Log-rank test. Cox regression analyses with relative risk (RR) with 95% confidence interval (CI) were used to estimate the association between clinical-pathological factors, nuclear BQ score, nuclear AR score, nuclear CCRK, and survival. The proportional-hazards assumption was examined using the Omnibus test, and no violation was observed: *, **, ***, and **** in the figures indicated *p* < 0.05, *p* < 0.01, *p* < 0.001, and *p* < 0.0001, respectively. The graphic abstract was created using BioRender (https://www.biorender.com/).

## 3. Results

### 3.1. Doxorubicin (DOX) Resistance Mediated by BQ Overexpression Was AR-Dependent

Androgen receptor (AR) activity has been shown to confer anastrozole resistance in ER+ve breast cancer, the effect being BQ-dependent [32]. We have previously shown that BQ overexpression confers resistance to anthracycline-based treatment [33]. Anthracycline suppresses the activity of DNA topoisomerase, resulting in the generation of double-strand DNA breakage. It has been shown that AR activation can regulate a network of DNA repair genes in prostate cancer [17]. Expression of constitutively active AR has been shown to modulate DNA repair and confer resistance to docetaxel [43,44]. Modulation of DNA repair mechanisms impacts the efficacy of DNA damage-inducing agents in cancer treatment [45]. Doxorubicin (DOX) is a commonly used anthracycline-based drug. Since BQ overexpression can enhance AR-signaling [32], we hypothesized that BQ may employ AR to induce resistance to DOX in ER+ve breast cancer by enhancing its DNA repair capacity.

**Figure 1:**
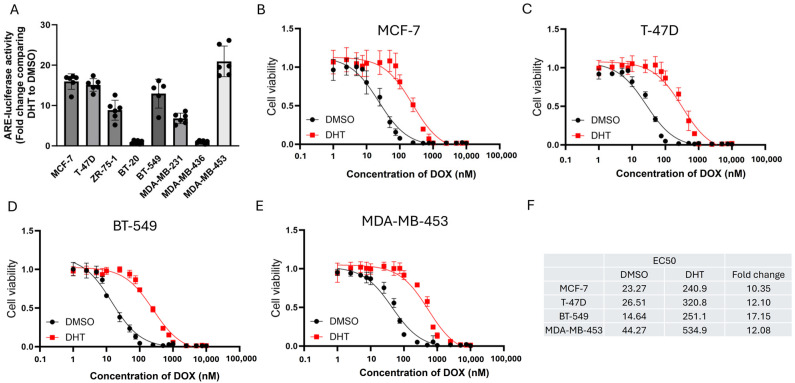
AR activation induced resistance to DOX. (**A**) AR activity in various breast cancer cell lines. Luciferase reporter assay with androgen-responsive element was employed to measure response to 1 nM of DHT. DMSO only was recorded as a baseline. The fold change of ARE was determined by comparing the expression levels between DHT and DMSO. Results were shown as mean ± SD from 5 independent experiments. The effect of AR activation induced by 1 nM of DHT on the response to various concentrations of DOX treatment in (**B**) MCF-7, (**C**) T-47D, (**D**) BT-549, and (**E**) MDA-MB-453. Results were shown as mean ± SD from 4 independent experiments. (**F**) EC50 values of DOX of each cell line in the presence and absence of DHT.

**Figure 2:**
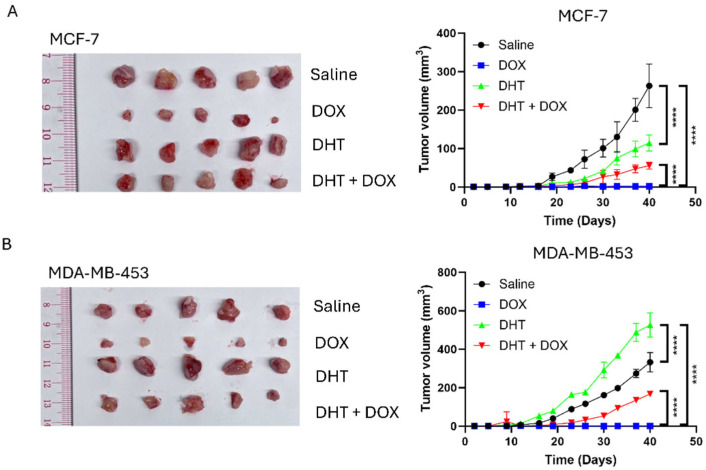
AR activation conferred resistance to DOX in vivo. Xenograft models were established using (**A**) MCF-7 and (**B**) MDA-MB-453 cell lines. 15 mg/Kg of DOX and 12.3 mg/Kg of DHT were used. The drugs were administered via subcutaneous injection. 2 treatments were performed each week. The graphs showed changes in tumor volume over time. Results were shown as mean ± SD from 5 mice. Two-way ANOVA was employed to determine the statistical significance. **** represents *p* < 0.0001.

**Figure 3:**
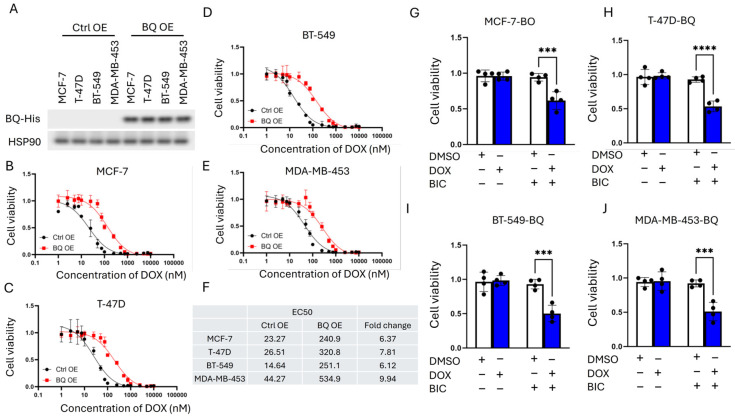
The effect of BQ overexpression on DOX resistance was AR dependent. (**A**) Overexpression of BQ in various breast cancer cell lines. Stable BQ overexpression cell lines were established, and expression of BQ was confirmed by Western blot with anti-HIS tag antibody. HSP90 was the loading control. Comparing response to DOX between the control (Ctrl OE) and BQ overexpression (BQ OE) in (**B**) MCF-7, (**C**) T-47D, (**D**) BT-549, and (**E**) MDA-MB-453. (**F**) EC50 values of DOX of each cell line in the presence (BQ OE) and absence (Ctrl OE) of BQ overexpression. AR suppression by 5 µM of BIC could reverse DOX resistance in (**G**) MCF-7-BQ, (**H**) T-47D-BQ, (**I**) BT-549-BQ, and (**J**) MDA-MB-453-BQ. Blue bars: DOX-treated; white bars: DOX-naive controls. Labels indicate treatment with 5 µM BIC and/or 50 nM of DOX. Results were shown as mean ± SD from 4 independent experiments. Each dot represents results from one independent experiment. Two-way ANOVA was employed to determine the statistical significance. *** and **** represent *p* < 0.001 and *p* < 0.0001, respectively.

First, we examined a panel of eight breast cancer cell lines, comprising both ER+ve (MCF-7, T-47D, ZR-75-1) and ER-ve (BT-20, BT-549, MDA-MB-231, MDA-MB-436, and MDA-MB-453) cell lines. The cells were transfected with a luciferase reporter containing an androgen-response element (ARE) and then treated with 1 nM of DHT. The fold change of ARE was determined by comparing DHT to DMSO. The results showed that both ER+ve (MCF-7, T-47D, ZR-75-1) and ER-ve (BT-549, MDA-MB-231 and MDA-MB-453) cell lines could respond to DHT (Figure 1A), while BT-20 and MDA-MB-436 barely responded to DHT (Figure 1A). Hence, the cell lines that demonstrated better response to DHT, namely MCF-7, T-47D, BT-549, and MDA-MB-453, were selected for further studies. The response of these cells to DOX was examined in the presence and absence of DHT (Figure 1B–E). From the curves, EC50 values were determined and shown in Figure 1F. In general, DHT treatment resulted in a shift in the curve to the right, with a greater than 10-fold increase in DOX resistance (Figure 1F). The in vivo effect of DHT on DOX response in MCF-7 and MDA-MB-453 xenografts confirmed these findings but also showed differences in response to treatment by DHT alone. As expected, DHT could not modulate the response to DOX in BT-20 and MDA-MB-436 (Figure S1). These results supported the importance of AR expression. Whilst DHT treatment alone suppressed MCF-7 tumor growth (Figure 2A), the growth of MDA-MB-453 was enhanced (Figure 2B). DOX resistance, however, was enhanced on AR activation in both models, as reflected by the increased tumor volume compared between DOX and DHT + DOX groups (Figure 2A,B). These findings confirm that AR activation induced DOX resistance in AR-responsive breast cancer cell lines. Since BQ overexpression has been shown to enhance AR-signaling [32], BQ expression may be responsible for the modulation of AR that confers DOX resistance. We established stable cell lines with BQ overexpression (BQ OE) and the control with empty vector (Ctrl OE) (Figure 3A). The response to DOX in these cell lines was examined (Figure 3B–E). EC50 values confirmed that BQ overexpression could induce resistance to DOX (Figure 3F). Indeed, cell viability assays showed that DOX resistance mediated by BQ overexpression could be compromised by 5 µM of BIC (Figure 3G–J). The results support our hypothesis that BQ overexpression modulates AR to confer DOX resistance.

### 3.2. BQ Overexpression Enhanced DNA Repair Capacity

**Figure 4:**
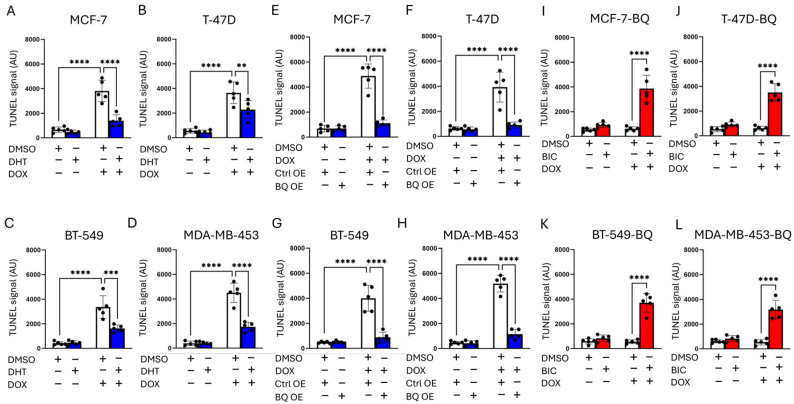
AR activity modulated the degree of DNA damage induced by DOX. DNA damage was assessed by the TUNEL assay. Activation of AR by 1 nM of DHT reduced the degree of DNA damage in (**A**) MCF-7, (**B**) T-47D, (**C**) BT-549, and (**D**) MDA-MB-453. Blue bars: 1 nM DHT-treated; white bars: DHT-naive. BQ overexpression compromised the effect of DOX on DNA damage induction in (**E**) MCF-7, (**F**) T-47D, (**G**) BT-549, and (**H**) MDA-MB-453. Blue bars: BQ overexpression (pcDNA3.1-His-BQ); white bars: control overexpression (pcDNA3.1-His). Suppression of AR by 5µM of BIC recovered the effect of DOX on the induction of DNA damage in (**I**) MCF-7-BQ, (**J**) T-47D-BQ, (**K**) BT-549-BQ, (**L**) MDA-MB-453-BQ. Red bars: 5 µM BIC-treated; white bars: BIC-naive. All panels used 50 nM DOX. Results were shown as mean ± SD from 5 independent experiments. Each dot represents results from one independent experiment. Two-way ANOVA was employed to determine the statistical significance. **, *** and **** represent *p* < 0.01, *p* < 0.001 and *p* < 0.0001, respectively.

**Figure 5:**
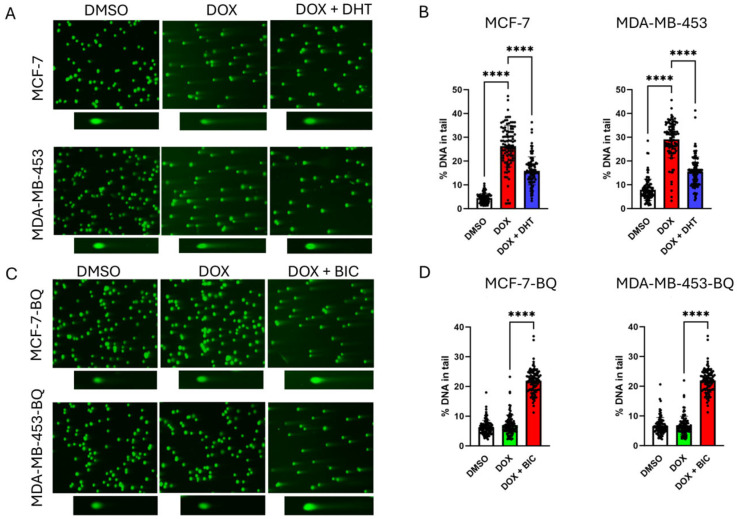
Modulation of AR activity interfered with the induction of DNA breakage. Comet assay was performed. (**A**) AR activation by DHT treatment reduced the degree of DNA damage. (**B**) Statistical analysis of A. (**C**) AR inhibition by treatment with BIC on BQ overexpressing cells sensitized the cells to DOX. Representative comet assay images are shown for each treatment condition, illustrating DNA damage as % DNA in the tail. (**D**) Statistical analysis of C. 50 nM of DOX, 5 µM of BIC, and 1 nM of DHT were used. Results were shown as mean ± SD from 100 nuclei. Each dot represents results from one nucleus. One-way ANOVA was employed. **** represents *p* < 0.0001.

Through a TUNEL assay, we confirmed that activation of AR by DHT treatment reduced the degree of DNA damage (Figure 4A–D). BQ OE reduced DOX-induced DNA damage (TUNEL signal) in MCF-7, T-47D, BT-549, and MDA-MB-453 (Figure 4E–H), and this effect was reversed by 5 µM BIC (Figure 4I–L), indicating AR-dependent DNA repair (Figure 4I–L). Comet assay at 48 h post-treatment with 50 nM DOX showed similar findings, confirming that DHT could reduce the degree of DNA damage induced by DOX in MCF-7 and MDA-MB-453 (Figure 5A,B). A similar assay performed on BQ overexpressing cells treated with DOX together with BIC at 48 h post-treatment with 50 nM of DOX, confirmed that suppression of AR activation by BIC treatment sensitized BQ overexpressing cells to DOX treatment, as indicated by the increased proportion of DNA in the tail (Figure 5C,D). However, initial DNA damage levels (e.g., at 24 h) were not assessed, limiting our ability to confirm equivalent primary lesion formation. These findings are strongly supportive that BQ expression effectively modifies AR activation, reducing the effect of DOX on DNA damage induction.

**Figure 6:**
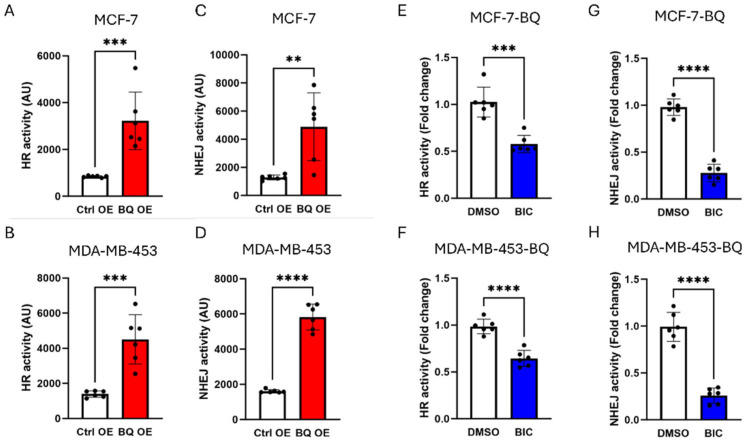
BQ employed AR to modulate HR and NHEJ activity. Overexpression of BQ enhanced HR activity in (**A**) MCF-7, (**B**) MDA-MB-453. Overexpression of BQ enhanced NHEJ activity in (**C**) MCF-7, (**D**) MDA-MB-453. Inhibition of AR with BIC suppressed HR activity in (**E**) MCF-70-BQ and (**F**) MDA-MB-453-BQ. Inhibition of AR with BIC suppressed NHEJ activity in (**G**) MCF-70-BQ and (**H**) MDA-MB-453-BQ. 5 µM of BIC was used. pDR-GFP assay was employed to monitor HR activity, while EJ5-GFP was used to monitor NHEJ activity. Results were shown as mean ± SD from 6 independent experiments. Each dot represents results from one independent experiment. Students’ *t*-test was used. **, *** and **** represent *p* < 0.01, *p* < 0.001 and *p* < 0.0001, respectively.

To determine whether the BQ-AR axis modulates the DNA repair mechanism in suppressing the effect of DOX, DR-GFP [35] and EJ5-GFP [36] assays were used to monitor DNA repair via homologous recombination (HR) and non-homologous end joining (NHEJ), respectively. BQ overexpression could enhance both HR and NHEJ activity in MCF-7 and MDA-MB-453 (Figure 6A–D). On inhibition of AR by 5 µM BIC, HR and NHEJ activity were reduced in the BQ overexpressing cells (Figure 6E–H). However, it was observed that HR activity was only reduced by half, whilst the effect on NHEJ was much more pronounced, with inhibition greater than 50%. These findings suggest that AR contributes significantly to NHEJ in BQ-overexpressing breast cancer cells.

### 3.3. Identification of CCRK as a Downstream Modulator of AR

**Figure 7:**
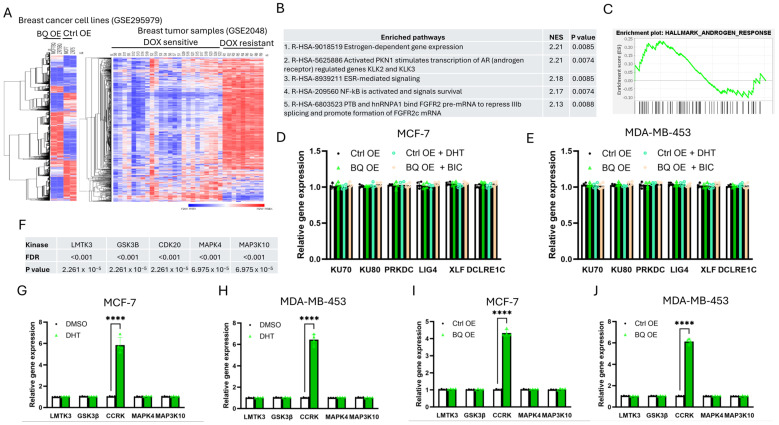
Identification of CCRK as a potential modulator of NHEJ. (**A**) Differential gene expression of GSE295979 and GSE2048 datasets. A heatmap was employed to show the pattern of differential gene expression. Red colour represents up-regulation while blue colour represents down-regulation. (**B**) 5 commonly enriched pathways. KEGG pathway enrichment analysis was performed using the GSE295979 and GSE2048 datasets. The lists of enriched pathways from each of the datasets were compared, and common pathways were identified. (**C**) Androgen response was found to be enriched using gene set enrichment analysis. Modulation of BQ and AR activity did not affect the mRNA level of NHEJ-related genes in (**D**) MCF-7 and (**E**) MDA-MB-453. Results were shown as mean ± SD from 4 independent experiments. (**F**) The differential gene expression from the two datasets suggested 5 kinases were potentially enhanced. AR activation by 1 nM of DHT enhanced CCRK expression in (**G**) MCF-7 and (**H**) MDA-MB-453. Overexpression of BQ enhanced CCRK expression in (**I**) MCF-7 and (**J**) MDA-MB-453. Results were shown as mean ± SD from 4 independent experiments. Each dot represents results from one independent experiment. Students’ *t*-test was used. **** represents *p* <0.0001.

RNA-sequencing (GSE295979) had previously been performed to determine the differential gene expression profile of BQ overexpressing cells [39]. From the public dataset (cDNA array; GSE2048) [38], we found differential gene expression results between DOX-sensitive and DOX-resistant breast tumors (Figure 7A). Comparing the differentially expressed genes from the two datasets, we identified commonly enriched pathways, with the top 5 pathways as shown in Figure 7B. By gene set enrichment analysis, AR signaling was suggested to be important (GSEA; Figure 7C). As we did not identify any pathway related to DNA repair, we determined by qPCR the expression of key members of NHEJ, including KU70, KU80, PRKDC (DNA-PKcs), LIG4 (DNA ligase 4), XLF, and DCLRE1C (ARTEMIS), and found that none of their mRNA was altered in the AR-activated cells, BQ-overexpressing cells. (Figure 7D,E). Furthermore, the addition of BIC could not alter the gene expression (Figure 7D,E). Therefore, we speculated that the BQ-AR axis employed an indirect method to modulate NHEJ activity. From the gene expression study (Figure 7A), we identified 5 kinases, whose activities were potentially enhanced (*p* < 0.001 and FDR < 0.001). On examination of their expression levels by qPCR, CCRK was the only candidate that was consistently enhanced on DHT treatment (Figure 7G,H) and in BQ overexpressing cells (Figure 7I,J).

**Figure 8:**
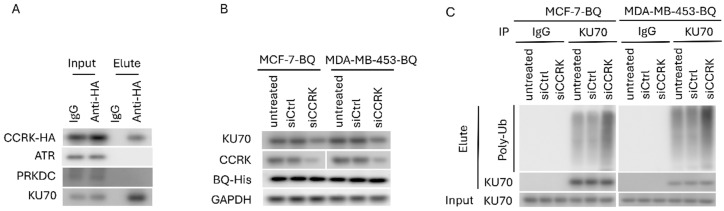
CCRK interacted with KU70 and modulated its protein stability. (**A**) CCRK was able to interact with KU70. MCF-7 was transfected with pcDNA3.1-HA-CCRK. CoIP was performed with anti-HA antibody 48 h post-transfection. Western blot was performed to determine the presence of the protein candidates in the immunoprecipitant. (**B**) Knockdown of CCRK reduced the expression of KU70 in the BQ overexpressing cells. MCF-7-BQ and MDA-MD-453-BQ were treated with 50 nM of the siRNA. Western blot was performed. GAPDH was the loading control. (**C**) Knockdown of CCRK enhanced poly-ubiquitination (poly-Ub) level on KU70. 5 µM of MG132 was used to inhibit proteasome. KU70 proteins were immunoprecipitated, and the level of Ub was analyzed by Western blot.

To examine whether CCRK would associate with NHEJ-related proteins, CoIP was performed. Results showed that CCRK could bind to KU70 but not the other candidate tested (Figure 8A; Figure S2). Knockdown of CCRK led to the reduction in KU70 expression in the BQ overexpressing cells (Figure 8B; Figure S3). Therefore, we speculated that KU70 might be a substrate of CCRK and CCRK might modulate the phosphorylation of KU70, which might interfere with protein stability. We confirmed that modulation of CCRK expression did not alter the mRNA expression of KU70 (Figure S4). Through ubiquitination assay, we confirmed that knockdown of CCRK could enhance the level of poly-ubiquitination (Poly-Ub) on KU70 (Figure 8C; Figure S5). Poly-Ub is a functional tag that directs a protein for degradation through the 26S proteasome [46]. Knockdown of CCRK reduced KU70 protein levels and increased polyubiquitination (Figure 8B,C), suggesting that CCRK might contribute to KU70 stability, potentially via post-transcriptional regulation, though the precise mechanism remains to be elucidated.

### 3.4. Clinical Significance of AR, BQ, and CCRK in ER-Positive Breast Cancer

**Figure 9:**
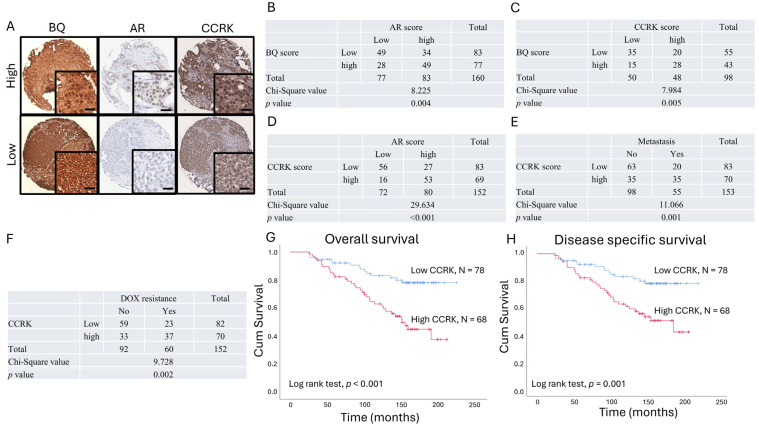
Clinical significance of CCRK in ER-positive breast cancer. (**A**) Expression of BQ, AR, and CCRK was assessed by immunohistochemistry. The scale bar represents 20 µm. H-score was employed to determine the nuclear expression of these proteins, and expression > median (cut-off) was regarded as a high expression level. The cut-offs for BQ, AR, and CCRK were 150, 105, and 120, respectively. Representative images were shown. Correlation between (**B**) AR and BQ, (**C**) CCRK and BQ, (**D**) CCRK and AR, (**E**) CCRK and metastasis, and (**F**) CCRK and DOX resistance. Chi-square test was employed. Patients with high expression of CCRK had poorer outcomes in terms of (**G**) overall survival and (**H**) disease-specific survival.

To support our findings in vivo, immunohistochemistry was performed on 170 primary breast tumor samples from ER+ve breast cancer patients (Table 1). Nuclear expression of BQ, AR, and CCRK was assessed. The cases were categorized as high and low expression, using the median score as the cut-off value (Figure 9A). BQ was found to be positively correlated with AR (Figure 9B; *p* = 0.004), consistent with the findings from our previous study [32] and also positively correlated with CCRK (Figure 9C; *p* = 0.005) using chi-square test. AR was found to be associated with CCRK (Figure 9D; *p* < 0.001). Next, we found that high expression of CCRK was associated with metastasis (Figure 9E; *p* = 0.001) and with DOX resistance (Figure 9F; *p* = 0.002). The Kaplan–Meier curve confirmed that patients with high CCRK expression had poorer overall survival outcomes (Figure 9G; *p* < 0.001) and disease-specific survival (Figure 9H; *p* < 0.001. Univariate Cox-regression analysis (Table 2) indicated that high expression of CCRK (*p* < 0.001; RR 3.056, 95% CI 1.661, 5.621, AR (*p* < 0.001; RR 3.420, 95% CI 1.783, 6.562), and BQ (*p* = 0.009; RR 2.175, 95% CI 1.210, 3.911) were associated with poorer overall survival outcomes. Univariate cox-regression analysis (Table 3) indicated that high expression of CCRK (*p* < 0.001; RR 2.731, 95% CI 1.472, 5.067), AR (*p* < 0.001; RR 3.092, 95% CI 1.601, 5.970) and BQ (*p* = 0.013; RR 2.145, 95% CI 1.172, 3.928) were also associated with poorer disease-specific survival outcomes.

## 4. Discussion

This study elucidates the role of BQ323636.1 (BQ), a splice variant of NCOR2, in conferring doxorubicin (DOX) resistance in ER+ breast cancer through an AR-dependent mechanism. Doxorubicin, an anthracycline chemotherapy agent, has the ability to induce DNA double-strand breaks (DSBs) by inhibiting topoisomerase II and generating reactive oxygen species [47] and is commonly used as adjuvant treatment, particularly for triple-negative breast cancer (TNBC) and metastatic ER+ve cases. It is frequently used in combination regimens, such as AC (doxorubicin and cyclophosphamide) or TAC (docetaxel, doxorubicin, and cyclophosphamide), to improve outcomes in both early-stage and metastatic settings [48]. However, drug resistance significantly undermines DOX efficacy, contributing to treatment failure and cancer-related mortality. Drug resistance is implicated in almost all cancer deaths in patients receiving chemotherapy, as resistant cancer cells evade apoptosis or repair drug-induced DNA damage, leading to disease progression and poor survival, particularly in aggressive subtypes like TNBC [6,49]. Our findings demonstrate that BQ overexpression enhances AR signaling, which upregulates cell cycle-related kinase (CCRK) (Figure 7G–J), contributing to stabilizing KU70 (Figure 8C), a critical component of non-homologous end joining (NHEJ) DNA-repair [50]. However, one important consideration should be noted regarding the poly-ubiquitination detected in Figure 8C, which was demonstrated using cell lysates where KU70 naturally associates with other protein factors. Therefore, the observed changes in the poly-ubiquitination signal might not be attributed exclusively to KU70 modifications. To strengthen these findings, ubiquitination assays using purified recombinant proteins and anti-K48-linked poly-ubiquitin antibodies could be performed to specifically address the direct effect of CCRK on KU70 poly-ubiquitination. Moreover, pulse-chase experiments could be conducted to further confirm KU70 protein stability and directly measure its half-life under different experimental conditions. AR-CCRK cascade enhances DNA repair capacity, reducing the efficacy of DOX-induced DNA damage, thus promoting chemoresistance. To further validate our findings, ionizing radiation (IR) could be employed instead of DOX. If the BQ–AR axis similarly enhances DNA repair following IR exposure, this would provide independent evidence of its role in modulating DNA repair mechanisms. Our observations are in keeping with results from prior studies showing the role of AR in modulating DNA repair pathways in prostate cancer [17,43,44,51,52], thus extending its relevance to ER+ve breast cancer. The absence of protein expression data for LMTK3, MAPK4, and MAP3K10, due to antibody unavailability, limits our understanding of their roles in the BQ-AR-CCRK-KU70 axis. Future studies will validate their protein expression and kinase activity in response to DHT to clarify their potential contributions to DOX resistance.

Whilst BQ overexpression enhanced both NHEJ and homologous recombination (HR) activities, we showed that upon AR inhibition with bicalutamide (BIC), NHEJ was more significantly reduced (>50%) than HR (~50%) (Figure 6E–H), suggesting the dominance of NHEJ in our breast cancer model. One limitation of our study is that neither the DR-GFP nor the EJ5-GFP assays included an internal reporter (e.g., mCherry or DsRed) to identify successfully transfected cells. As a result, transfection efficiency may have varied between samples. The lower TUNEL signal in BQ OE cells (Figure 4E–L) reflects enhanced DNA repair, with BIC restoring damage sensitivity, consistent with the BQ-AR axis enhancing NHEJ. The comet assay results (Figure 5) support enhanced NHEJ-mediated repair in BQ-overexpressing cells, but the lack of early time point data (e.g., 24 h) or markers like γH2AX or phospho-ATM (S1981) precludes confirmation of comparable initial DNA damage levels. This limits our ability to distinguish whether reduced DNA damage reflects enhanced repair kinetics or altered damage sensing. Future studies will measure early DNA damage markers to clarify this mechanism. The contribution of HR, potentially via AR-mediated RAD51 regulation [23], to DOX resistance remains underexplored, and HR-specific assays (e.g., RAD51 knockdown) could serve to clarify its role in the future. Further experiments are also needed to determine whether the prominence of NHEJ is cell-line-specific. The role of CCRK in stabilizing KU70 is novel, as confirmed by co-immunoprecipitation and increased KU70 polyubiquitination upon CCRK knockdown (Figure 8B,C). This suggests the involvement of post-transcriptional regulation, since the NHEJ gene mRNA levels were unchanged (Figure 7D,E). However, the precise mechanism of KU70 stabilization requires further study.

The identification of CCRK as a downstream mediator of AR signaling is a novel finding. The interaction of CCRK with KU70, enhancing its phosphorylation and stability, suggests a previously unrecognized mechanism by which AR modulates NHEJ. This is particularly relevant given that KU70 is essential for NHEJ-mediated repair of DSBs [18,20]. However, the lack of significant upregulation of NHEJ-related gene mRNA levels, including KU70, in BQ-overexpressing cells (Figure 7D,E) suggests post-transcriptional regulation. While we confirmed increased polyubiquitination upon CCRK knockdown (Figure 8C), direct evidence of specific phosphorylation sites on KU70 targeted by CCRK is lacking. This limits the mechanistic clarity of how BQ regulates KU70 protein levels. Future experiments, such as mass spectrometry to identify KU70 phosphorylation sites or pulse-chase assays to measure KU70 protein stability, are needed to substantiate this mechanism. While our data suggest CCRK stabilizes KU70, the lack of K48-linkage analysis and potential ubiquitination of KU70-binding partners limit mechanistic clarity. Future studies using co-IP under denaturing conditions and cycloheximide chase experiments will confirm whether CCRK directly modulates KU70 half-life. Additionally, co-immunoprecipitation studies to confirm a BQ-AR-CCRK-KU70 complex could strengthen the proposed pathway. While our study focused on DOX, other DSB-inducing agents (e.g., bleomycin, ionizing radiation) are expected to show similar BQ-mediated resistance via NHEJ, whereas non-DSB treatments (e.g., taxanes, nucleotide analogs) may remain unaffected. Future studies testing these agents will clarify the specificity of the BQ-AR-CCRK-KU70 axis.

Clinically, analyzing expression by IHC on 170 primary tumor samples of ER+ve breast cancer patients who received DOX neoadjuvant/adjuvant chemotherapy, we observed a positive correlation between high BQ, AR, and CCRK expression, which correlated with poorer prognosis, as well as DOX resistance (Figure 9), in keeping with our previous findings reporting the role of BQ in epirubicin and anastrozole resistance [32,33]. Our data, however, require further validation using larger patient cohorts with survival analyses. Further studies should elucidate how BQ integrates the various resistance mechanisms across different therapeutic classes. The differential DOX response in AR-responsive cell lines (e.g., MCF-7, T-47D, BT-549, MDA-MB-453) versus non-responsive lines (e.g., BT-20, MDA-MB-436) supports AR as a potential biomarker for predicting DOX sensitivity, with BIC abolishing BQ-mediated resistance in vitro and in vivo, highlighting therapeutic potential.

The inclusion of ER-negative cell lines (e.g., BT-549, MDA-MB-231, MDA-MB-453) in our study, despite the focus on ER+ breast cancer, was intended to validate AR responsiveness to DHT across subtypes (Figure 1A). However, this raises concerns about overgeneralization, as ER-negative subtypes have distinct biology. While these cell lines demonstrated AR-dependent DOX resistance, the findings may not be fully applicable to ER+ve breast cancer. Future studies should prioritize ER+ve models or delineate subtype-specific effects to avoid misinterpretation. Additionally, the dual role of AR as a tumor suppressor in ER+ve breast cancer [28] and a contributor to DOX resistance via DNA repair poses a logical complexity. We hypothesize that the tumor-suppressive effect of AR (e.g., inhibiting ER signaling) is distinct from its DNA repair functions, which are amplified by BQ overexpression. This duality necessitates subtype-specific therapeutic strategies, as AR inhibition may have varying effects in ER+ versus ER-/HER2+ contexts.

Therapeutically, combining BIC with DOX or targeting CCRK could be used to overcome DOX resistance in ER+ve/AR+ve patients with high BQ expression. However, given the role of CCRK in other cancers [53,54,55], suggests caution for off-target effects. Limitations include reliance on cell lines, which may not fully reflect tumor complexity, and the lack of exploration of crosstalk with pathways such as PI3K/AKT [12,13]. Validation in patient-derived xenografts and studies of additional pathways are also needed. In conclusion, the BQ-AR-CCRK-KU70 axis drives DOX resistance in ER+ breast cancer through NHEJ, providing a foundation for personalized therapies that combine AR/CCRK inhibitors with DOX.

## 5. Conclusions

This study establishes BQ as a key modulator of DOX resistance in ER+ve breast cancer through an AR-CCRK-KU70 axis that enhances NHEJ-mediated DNA repair. While promising, the findings require further mechanistic and clinical validation to address limitations and refine therapeutic approaches. These insights provide a foundation for personalized treatments combining AR or CCRK targeting with DOX to improve outcomes in patients with high BQ expression.

## Figures and Tables

**Table 1:** Clinical characterization of breast cancer patients.

Clinical Characters		Number of Cases	Percentage (%)
ER-positive breast cancer patients		170	100
Age	<50	86	50.6
	≥50	77	45.3
T stage	I, II	146	85.9
	III, IV	17	10.0
Lymph Node status	Positive	133	78.2
	Negative	35	20.6
Tumor Grade	1, 2	119	70.0
	3	40	23.5
Tumor Size	<2 cm	13	7.6
	≥2 cm	117	68.8
Progesterone receptor status	Positive	125	73.5
	Negative	44	25.9
HER2 receptor status	Positive	26	15.3
	Negative	106	62.4
CCRK nucleus score	Positive	153	90.0
	Negative	0	0.0
AR nucleus score	Positive	152	89.4
	Positive	163	95.9
BQ nucleus score	Positive	163	95.9
	Negative	0	0.0

**Table 2:** Cox regression analyses overall survival in ER+ve breast cancer.

Clinical-Pathological Parameters	Univariate Analysis	
	RR (95% CI)	*p* value
Age (N = 163)	1.335 (0.781, 2.283)	0.290
T-stage (N = 158)	2.393 (1.161, 4.931)	0.018
Lymph-node involvement (N = 162)	2.931 (1.165, 7.376)	0.022
Tumor-Grade (N = 154)	1.768 (0.994, 3.142)	0.052
Histological type (N = 157)	1.003 (0.427, 2.354)	0.995
PR status (N = 162)	0.545 (0.314, 0.948)	0.031
HER2 status (N = 127)	1.975 (1.007, 3.871)	0.048
Tumor size (N = 126)	1.507 (0.463, 4.912)	0.496
Cases with high CCRK nucleus score (N = 146)	3.056 (1.661, 5.621)	<0.001
Cases with high AR nucleus score (N = 155)	3.420 (1.783, 6.562)	<0.001
Cases with high BQ nucleus score (N = 156)	2.175 (1.210, 3.911)	0.009

**Table 3:** Cox regression analyses disease-specific survival in ER+ve breast cancer.

Clinical-Pathological Parameters	Univariate Analysis	
	RR (95% CI)	*p* value
Age (N = 163)	1.212 (0.694, 2.117)	0.499
T-stage (N = 158)	2.604 (1.255, 5.403)	0.010
Lymph-node involvement (N =1 62)	2.633 (1.042, 6.651)	0.041
Tumor-Grade (N = 154)	1.839 (1.013, 3.339)	0.045
Histological type (N = 157)	0.916 (0.388, 2.161)	0.841
PR status (N = 162)	0.524 (0.296, 0.928)	0.027
HER2 status (N = 127)	1.812 (0.905, 3.629)	0.093
Tumor size (N = 126)	1.303 (0.397, 4.274)	0.662
Cases with high CCRK nucleus score (N = 146)	2.731 (1.472, 5.067)	<0.001
Cases with high AR nucleus score (N = 155)	3.092 (1.601, 5.970)	<0.001
Cases with high BQ nucleus score (N = 156)	2.145 (1.172, 3.928)	0.013

## Data Availability

The materials and resources in this study are available from the corresponding author upon reasonable request.

## Supplementary Materials

The following supporting information can be downloaded at: https://www.mdpi.com/article/10.3390/cells14171341/s1, Figure S1. DHT treatment did not modulate the response to DOX in BT-20 and MDA-MB-436. Figure S2. Quantification of Figure 8A. Figure S3. Quantification of Figure 8B. Figure S4. CCRK knockdown or overexpression did not affect mRNA level of KU70 in MCF-7-BQ and MDA-MB-453-BQ. Figure S5. Quantification of Figure 8C.
