# Supplementary material for: BQ323636.1 Employs the AR-CCRK Axis to Modulate the Expression of KU70 to Interfere with Non-Homologous End Joining Mediated DNA Repair Mechanism
Source: Cells. 2025 Aug 29;14(17):1341. doi: 10.3390/cells14171341 (PMC12428711; doi:10.3390/cells14171341)
Supplement: Supplementary file 1 [file cells-14-01341-s001.zip › cells-3732501-supplementary.pdf]

Supplementary figure legends

Figure S1

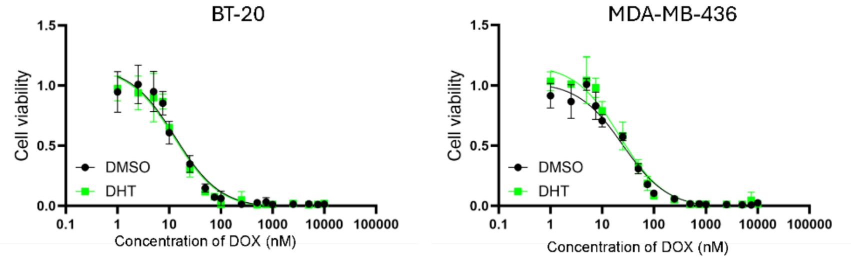

Fig. S1 DHT treatment did not modulate the response to DOX in BT-20 and MDA-MB-436. 1 nM of DHT was employed. The response to various concentrations of DOX treatment in BT-20 and MDA-MB-436 was determined using CCK8 assay. The cells were treated for 96 hours. Results were shown as mean ± SD from 4 independent experiments.

Figure S2

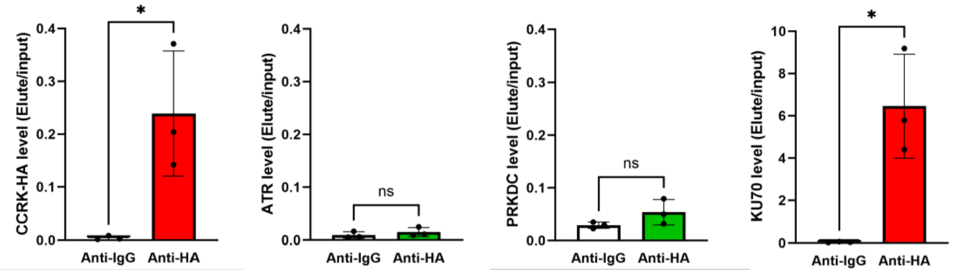

Fig. S2 Quantification of Fig. 8A. The ratio of elute to input was determined. Results were

shown as mean ± SD from 3 independent experiments. Students' t test was used. * represents $p < 0.05$.

Figure S3

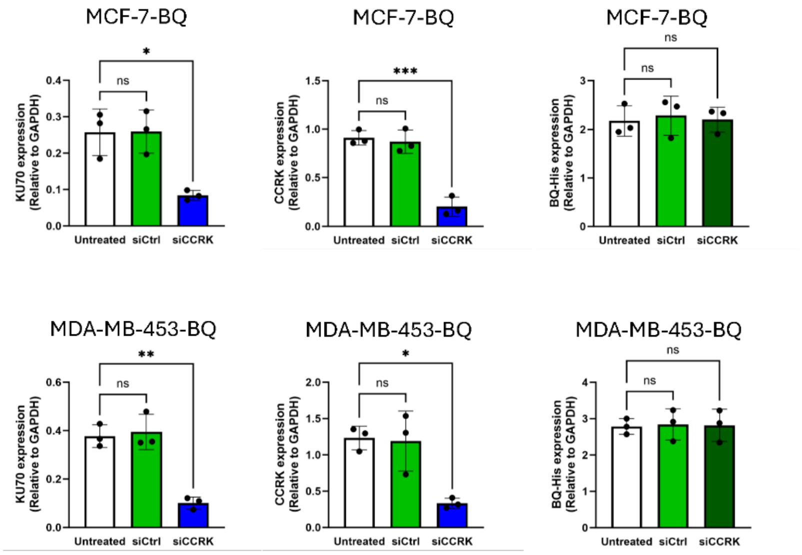

Fig. S3 Quantification of Fig. 8B. Protein expression relative to GAPDH was determined. Results were shown as mean ± SD from 3 independent experiments. One-way ANOVA was used. *, **, and *** represent $p < 0.05$, $p < 0.01$ and $p < 0.001$.

Figure S4

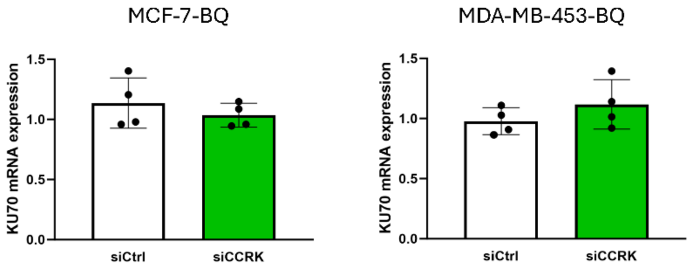

Fig. S4. CCRK knockdown or overexpression did not affect mRNA level of KU70 in

MCF-7-BQ and MDA-MB-453-BQ. qPCR was employed. Results were shown as mean ± SD from 4 independent experiments. Students' t test was used.

Figure S5

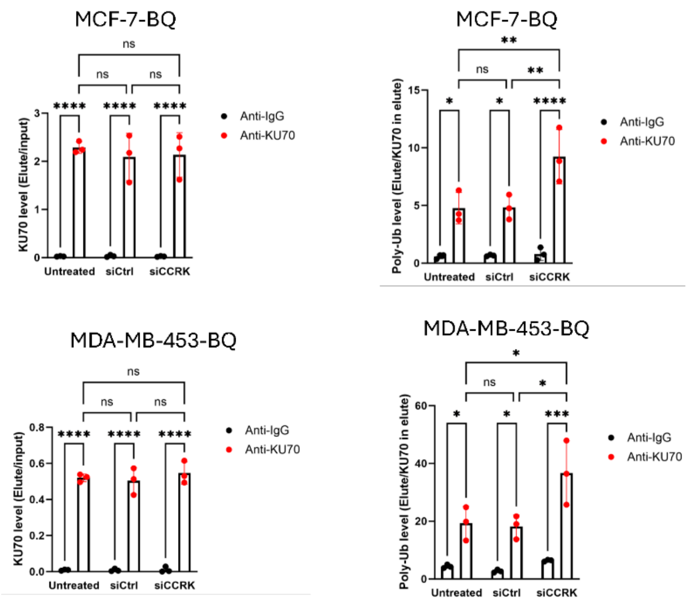

Fig. S5 Quantification of Fig. 8C. KU70 level was determined by determining the ratio of elute to input to obtain a normalised KU70 level in the elute. Poly-Ub level was assessed by determining the ratio of poly-Ub in elute to the normalised KU70. Results were shown as mean ± SD from 3 independent experiments. Two-way ANOVA was used. *, **, ***, and **** represent $p < 0.05$, $p < 0.01$, $p < 0.001$ and $p < 0.0001$.
